# Is There an Optimal Autonomic State for Enhanced Flow and Executive Task Performance?

**DOI:** 10.3389/fpsyg.2019.01716

**Published:** 2019-08-14

**Authors:** Michael S. Chin, Stefanos N. Kales

**Affiliations:** ^1^Division of General Internal Medicine and Public Health, Vanderbilt University School of Medicine, Nashville, TN, United States; ^2^Vanderbilt Occupational Health, Vanderbilt University Medical Center, Nashville, TN, United States; ^3^Environmental and Occupational Medicine and Epidemiology Program, Harvard T.H. Chan School of Public Health, Boston, MA, United States; ^4^Department of Occupational Medicine, Cambridge Health Alliance, Harvard Medical School, Boston, MA, United States

**Keywords:** flow, heart rate variability, cognitive performance, parasympathetic and sympathetic reactivity, sympathovagal balance

## Abstract

**Introduction:**

Flow describes a state of optimal experience that can promote a positive adaptation to increasing stress. The aim of the current study is to identify the ideal autonomic state for peak cognitive performance by correlating sympathovagal balance during cognitive stress with (1) perceived flow immersion and (2) executive task performance.

**Materials and Methods:**

Autonomic states were varied in healthy male participants (*n* = 48) using combinations of patterned breathing and skeletal muscle contraction that are known to induce differing levels of autonomic response. After autonomic variation, a Stroop test was performed on participants to induce a mild stress response, and autonomic arousal was assessed using heart rate variability. Subjective experience of flow was measured by standardized self-report, and executive task performance was measured by reaction time on the Stroop test.

**Results:**

There were significant associations between autonomic state and flow engagement with an inverted U-shaped function for parasympathetic stimulation, sympathetic response, and overall sympathovagal balance. There were also significant associations between autonomic states and reaction times. Combining sympathetic and parasympathetic responses to evaluate overall sympathovagal balance, there was a significant U-shaped relationship with reaction time.

**Discussion:**

Our results support the flow theory of human performance in which the ideal autonomic state lies at the peak of an inverted-U function, and extremes at either end lead to both suboptimal flow experience. Similarly, cognitive task performance was maximized at the bottom of the U-function. Our findings suggest that optimal performance may be associated with predominant, but not total, sympathetic response.

## Introduction

Recent public health focus on occupational burnout and stress resiliency has prompted further investigation into the role of the autonomic system in cognitive performance. Increasing number of recent studies have suggested that burnout is related to autonomic dysfunction during excessive stress (Lennartsson et al., 2016; Kanthak et al., 2017; May et al., 2018; Zhang et al., 2018; Traunmuller et al., 2019). However, despite these concerning observations, the effects of autonomic state on cognitive performance have not been fully defined, and some degree of increased stress may actually be desired for task performance. Increased autonomic arousal correlating with improved task performance is supported by several studies (Luft et al., 2009; Mathewson et al., 2010; Murray and Russoniello, 2012).

As a possible explanation resolving these seemingly conflicting findings, flow theory, as proposed by psychologist Csikszentmihalyi (1975), describes a state of optimal experience characterized by complete task immersion, effortless intention, intrinsic reward, and increased perception of control. This experience can promote a positive adaptation to increasing stress by a matching growth between challenges and skills through immediate task feedback.

The ideal physiological conditions to facilitate the flow experience have not been established. According to Csikszentmihalyi’s (1975) theory, the flow state lies on the continuum between boredom and anxiety. Csikszentmihalyi (1975) proposed that there were physiological changes associated with the flow experience, but recognized this association between physiology and psychology were not readily established. Previous studies have identified a possible association between increased sympathetic enhancement during the flow experience, but none have adequately demonstrated an optimal level of autonomic arousal for both task performance and subjective flow experience (Keller et al., 2011; Gaggioli et al., 2013; Peifer et al., 2014; Bian et al., 2016).

Many popular mind–body disciplines such as Yoga, Tai Chi, and Qigong, have been shown to activate the parasympathetic nervous system to varying degrees (Goyal et al., 2014; Sullivan et al., 2018; Walther et al., 2018). As part of a related project, the authors previously studied healthy male subjects’ responses to preconditioning using various rhythmic breathing and skeletal muscle contraction methods to vary their baseline autonomic states (Chin and Kales, 2019). Our previous study assessed paced respiration and dynamic tension through rhythmic skeletal muscle contraction as two core components common to Yoga, Tai Chi, and Qigong to better understand their interaction in activating the parasympathetic nervous system. The activation of the body’s parasympathetic nervous system has been demonstrated to occur through respiratory entrainment effects (Jerath et al., 2006; Nijjar et al., 2014) as well as voluntary rhythmic muscle contraction (Lehrer et al., 2009; Vaschillo et al., 2011).

The aim of the current study is to utilize the autonomic variability resulting from these different patterns of preconditioning, when faced with a cognitive stressor, to identify an autonomic state for maximized cognitive performance. This study examines the relationship between sympathovagal balance during cognitive stress with (1) perceived flow immersion, and (2) task performance. Sympathovagal balance was assessed by measurement of heart rate variability (HRV), which can be considered an indicator of autonomic activity (McCraty and Shaffer, 2015). A recent meta-analysis of 37 studies by Kim et al. (2018) concluded that HRV is impacted by stress and can be used as an objective assessment of psychological stress.

## Materials and Methods

### Participants

Forty-eight healthy male participants, ages 18–55 years were recruited from Harvard T.H. Chan School of Public Health- and Harvard Medical School-affiliated student programs, fellowships, and training residencies. Males were enrolled to minimize HRV due to hormonal variation (Sato and Miyake, 2004; Thayer et al., 2012; Koenig and Thayer, 2016). Any individuals with history of restrictive or obstructive lung disease, hypertension, or taking any medications that could affect heart rate were excluded. Caffeine consumption was not specifically restricted since withdrawal effects on HRV may exist for habitual caffeine users (Zimmermann-Viehoff et al., 2016).

### Procedure

The study protocol was reviewed and approved by the IRB of the Harvard T.H. Chan School of Public Health. Testing occurred over a single 30-min session between the daytime hours of 09:00 and 16:00. A Polar H7 heart rate monitor (Polar Electro Oy, Kempele, Finland) was used as a validated research device to measure R–R intervals with accuracy comparable to electrocardiograms (Barbosa et al., 2016; Giles et al., 2016). A logging application on iPad (Apple Inc., Cupertino, CA, United States) recorded the R–R interval signals from the chest strap which were further analyzed.

Participants sat upright quietly for 5 min while reading the instructions for the study, and then, heart rate, cuff blood pressure, and respiration rate were measured. To generate varying baseline autonomic states among participants, subjects were randomized to one of four preconditioning groups: (1) nasal respiration at 0.1 Hz (inhale nose 5 s, exhale mouth 5 s) for 5 min, (2) contracting arm muscles by grasping a tennis ball at 0.1 Hz for 5 min (alternating contractions in left and right arms every 5 s), (3) performing contraction and nasal respiration in synchrony at 0.1 Hz for 5 min, and (4) reading consecutively four articles rated as emotionally neutral for 5 min. The contraction tasks have been demonstrated to vary cardiac reactivity through differing levels of resonance (Lehrer et al., 2000, 2009; Vaschillo et al., 2011). A graphical timer application on iPad was used to visually cue the breathing/contraction. The articles were *Scientific American* excerpts that were previously validated as emotionally neutral (van den Broek et al., 2001).

To assess executive task function, a computerized version of the Stroop test^1^ was run for 5 min. The Stroop test has been demonstrated to produce a mild sympathetic response through dissonant executive task function (Salahuddin et al., 2007; Visnovcova et al., 2014). Participants were asked to indicate the color of the word (and not its meaning) by keystroke, as quickly as possible, while minimizing their errors. For congruent trials, the displayed word and the color described by the word were the same. For incongruent trials, the displayed word and color presented were not the same. The reaction time for each word pair is recorded by the computer program with the premise of the Stroop test that incongruent pairs have longer reaction times when compared to congruent pairs (Dyer, 1973). As a marker for performance, a reaction time gap was calculated for each participant from the difference between congruent and incongruent pair reaction times. A shorter reaction time gap was considered to indicate higher performance.

At the end of this task period, a 5-min questionnaire Short Flow State Scale-2 (SFSS-2) was then administered to assess degree of flow engagement (Jackson et al., 2008). Flow engagement is the degree of perceived task immersion, and the SFSS-2 has been validated for evaluation of performance engagement. Respiration rates were monitored during all phases of testing to ensure they were within the 9–24 cycles/min range required to correspond accurately to vagal tone (Laborde et al., 2017).

### Heart Rate Variability Analyses

Recorded R–R intervals were analyzed offline using Kubios HRV Premium software (Kubios Oy, Kuopio, Finland) using 2-min intervals based on recommendations from published standards (Task Force of the European Society of Cardiology and the North American Society of Pacing and Electrophysiology, 1996). Frequency domain measures analyze the power distribution of HRV as a function of high frequency (HF) and low frequency (LF). HF and LF components are reflective of parasympathetic and sympathetic activation, respectively. LF/HF can be regarded as the overall sympathovagal balance and degree of autonomic arousal (Pagani et al., 1986; Shaffer and Ginsberg, 2017).

**FIGURE 1:**
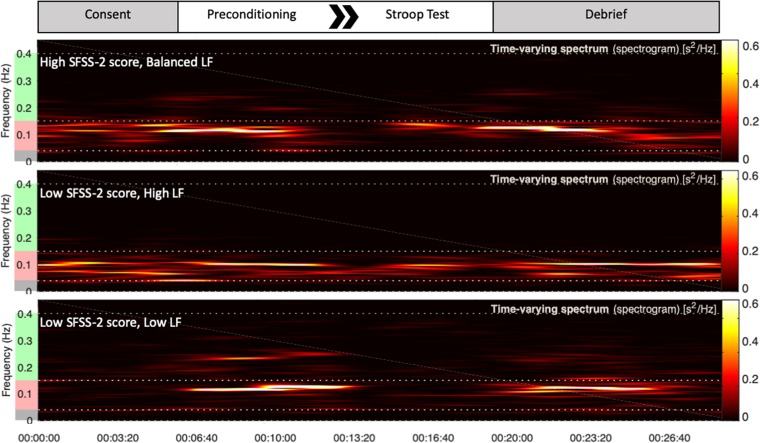
Top portion of the figure is a schematic of each study session. Underneath are representative examples of HRV frequency domain measures during the session for three different subjects. Increased power in the green frequency band represents parasympathetic HF response. Increased power in the red frequency band represents sympathetic LF response. During the Stroop test, the top subject exhibited high flow with a balanced LF response. The bottom two subjects, at extremes of either high or low sympathetic response, reported low flow.

Heart rate variability recordings during Stroop test were processed by Kubios HRV Premium. Automated artifact correction was performed for all recordings prior to analysis. One-hundred twenty second sampling periods were utilized to derive LF, HF, and LF/HF using Fast Fourier transformation spectrum method (Figure 1). LF and HF bands were standardly defined as 0.04–0.15 and 0.15–0.4 Hz, respectively, and absolute power for each band was analyzed in normalized units, LF or HF divided by total power (Malliani et al., 1994).

### Statistical Analyses

All statistical analyses were performed using Prism 8 (GraphPad Software, San Diego, CA, United States). To analyze the overall effects of varying HRV on reaction time, HRV measures (LF, HF, LF/HF) for all subjects during the Stroop test were regressed on measured reaction time using the least squares regression method, with no weighting. Both linear and quadratic models were generated for each comparison. For all regressions, assumption of homoscedasticity was made. To assess the effects of varying HRV on flow engagement, HRV measures were similarly regressed on SFSS-2 scores, testing both linear and quadratic models. *R*^2^-values for each relationship were evaluated at *p* < 0.05 significance level. Outliers were identified for each analysis using robust regression and outlier removal, which is a validated method of outlier detection included in Prism 8 (Motulsky and Brown, 2006). The outlier false discovery rate was set at <1%. Any reported outliers reported were excluded from the analysis.

## Results

All enrolled participants (*n* = 48) completed the study sessions without any adverse events. Participants’ average age was 29.9 years (*SD* ± 5.96). As a result of randomization, 12 subjects were each assigned to one of the four preconditioning groups.

### HRV Measurements Between Preconditioning Groups

A previous study compared LF, HF, and LF/HF responses between the preconditioning groups measured during application of the Stroop test (Chin and Kales, 2019). As a summary of the findings of this analysis, the alternating contraction group had 71.7% higher activation of parasympathetic signal over respiration alone (*p* < 0.001). Alternating contractions synchronized with breathing demonstrated 150% higher parasympathetic activation than control (*p* < 0.0001). Between contraction alone and synchronized contraction groups, the synchronized group demonstrated 45.9% higher parasympathetic response during the cognitive stressor (*p* < 0.001).

### HRV and Maximal Flow Engagement

**FIGURE 2:**
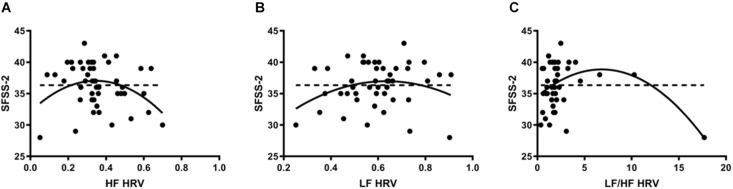
**(A)** Inverted U-shaped relationship between parasympathetic stimulation and SFSS-2 scores (*DF* = 44, *R*^2^ = 0.110, *p* < 0.0001); one outlier was identified and excluded from this analysis. Dotted line represents the linear model (*DF* = 46, *R*^2^ = 0.026, *p* > 0.05). Each point represents a single participant’s HF measured during the cognitive stressor. **(B)** A reciprocal relationship was found with an inverted U-shaped relationship between increasing sympathetic response (LF) and SFSS-2 scores (*DF* = 45, *R*^2^ = 0.070, *p* < 0.001). Dotted line represents the linear model (*DF* = 46, *R*^2^ = 0.00087, *p* > 0.05). Each point represents a single participant’s LF measured during the cognitive stressor. **(C)** When combining sympathetic and parasympathetic responses to measure overall sympathovagal balance, LF/HF demonstrated a significant inverted-U relationship when regressed on SFSS-2 (*DF* = 45, *R*^2^ = 0.187, *p* < 0.0001). The interpolated value of the vertex was LF/HF 6.822 with a maximal SFSS-2 score of 38.85. Dotted line represents the linear model (*DF* = 46, *R*^2^ = 0.031, *p* > 0.05). Each point represents a single participant’s LF/HF measured during the cognitive stressor.

Heart rate variability was regressed onto SFSS-2 scores to analyze autonomic association with flow engagement. Representative examples of HRV time varying frequency domain measures and flow scores are in Figure 1. In all cases, quadratic functions were statistically significant, whereas linear functions were not. HF (Figure 2A) indicated an inverted U-shaped relationship between parasympathetic stimulation and SFSS-2 scores (*DF* = 44, *R*^2^ = 0.110, *p* < 0.0001); one outlier was identified and excluded from this analysis. Similarly, when analyzing the sympathetic response (Figure 2B), a reciprocal relationship was found with an inverted U-shaped relationship between increasing and SFSS-2 scores (*DF* = 45, *R*^2^ = 0.070, *p* < 0.001). When combining sympathetic and parasympathetic responses to measure overall sympathovagal balance, LF/HF (Figure 2C) demonstrated a significant inverted-U relationship when regressed on SFSS-2 (*DF* = 45, *R*^2^ = 0.187, *p* < 0.0001). The interpolated value of the vertex was LF/HF 6.822 with a maximal SFSS-2 score of 38.85.

### HRV and Cognitive Performance

**FIGURE 3:**
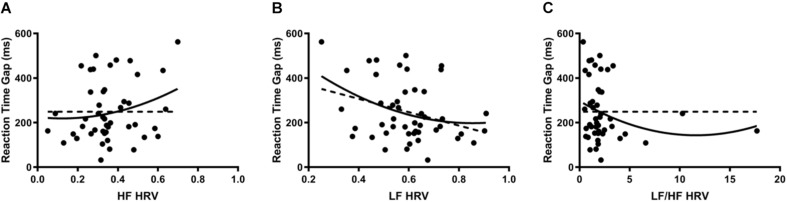
**(A)** When HRV was regressed onto reaction time gaps, HF indicated a positive curvilinear relationship between parasympathetic stimulation and reaction time (*DF* = 45, *R*^2^ = 0.053, *p* < 0.05). Dotted line represents the linear model (*DF* = 46, *R*^2^ = 0.046, *p* > 0.05). Each point represents a single participant’s HF measured during the cognitive stressor. **(B)** When analyzing the sympathetic response, a reciprocal relationship was found with increasing LF resulting in decreasing reaction time gaps (*DF* = 45, *R*^2^ = 0.117, *p* < 0.05). Dotted line represents the linear model (*DF* = 46, *R*^2^ = 0.10, *p* < 0.05). Each point represents a single participant’s LF measured during the cognitive stressor. **(C)** After combining sympathetic and parasympathetic responses to measure overall sympathovagal balance, LF/HF demonstrated a significant U-shaped relationship when regressed on reaction time (*DF* = 45, *R*^2^ = 0.046, *p* < 0.0001). The interpolated value of the vertex was LF/HF 11.61 with a minimal reaction time gap of 143.5 ms. Dotted line represents the linear model (*DF* = 46, *R*^2^ = 0.0083, *p* > 0.05). Each point represents a single participant’s LF/HF measured during the cognitive stressor.

Reaction time gaps were used as a marker for cognitive performance. When HRV was regressed onto reaction time gaps, there were significant second-order relationships for all measures; linear regression analysis demonstrated a significant relationship only for LF. HF (Figure 3A) indicated a positive curvilinear relationship between parasympathetic stimulation and reaction time (*DF* = 45, *R*^2^ = 0.053, *p* < 0.05). When analyzing the sympathetic response (Figure 3B), a reciprocal relationship was found with increasing LF resulting in decreasing reaction time gaps (*DF* = 45, *R*^2^ = 0.117, *p* < 0.05). After combining sympathetic and parasympathetic responses to evaluate overall sympathovagal balance, LF/HF (Figure 3C) demonstrated a significant U-shaped relationship when regressed on reaction time (*DF* = 45, *R*^2^ = 0.046, *p* < 0.0001). The interpolated value of the vertex was LF/HF 11.61 with a minimal reaction time gap of 143.5 ms.

## Discussion

After autonomic markers were regressed on flow scores, we demonstrated a possible inverted U-relationship between overall sympathovagal balance and self-reported experience of flow. With the exception of the LF relationship to reaction time, none of the linear models were statistically significant. Our finding supports Csikszentmihalyi’s (1975) theory of human performance in which the optimal autonomic state lies at the peak of an inverted-U, in which extremes at either end lead to suboptimal experience.

Studies on leisure activities, such as playing piano (de Manzano et al., 2010) and video games (Kozhevnikov et al., 2018), have effectively demonstrated the left side of the inverted-U curve, in which increasing sympathetic activation leads to increased flow experience. Paradoxically, other studies on job stress illustrate that increasing sympathetic arousal can also lead to declining performance and burnout. A recent systematic review identified 13 studies which confirmed a negative association between parasympathetic response and job stress or burnout (de Looff et al., 2018). When taken together, these seemingly opposing responses to arousal may actually just represent different sides of the inverted-U relationship demonstrated in our results.

A significant limitation in previous studies remained that the full spectrum of sympathetic arousal had not been represented due to insufficiently varied autonomic states. Peifer et al. (2014) addressed this shortcoming in study design by varying the arousal stimulus and inducing differing levels of social stress prior to task testing. Interestingly, using this design to create further autonomic variability, the authors did successfully demonstrate a quadratic inverted-U relationship for sympathetic LF HRV. However, they demonstrated only a linear association between increasing HF parasympathetic response and flow experience, but it should be noted that the study power was limited by the small study sample of only 22 participants.

Similar to Peifer et al. (2014)’s study design, we varied our baseline autonomic states. However, instead of priming subjects using varying social stressors, our methodology used differing combinations of patterned breathing and skeletal muscle contraction that are known to induce varying levels of autonomic response. With this preconditioning, we were able to prime enough baseline autonomic variation to demontrate the inverted-U relationship between increasing sympathetic arousal and flow experience. Improving on Peifer et al. (2014), we did successfully demonstrate an inverted-U relationship between increasing parasympathetic response and flow. Furthermore, when analyzing combined sympathic and parasympathetic balance as LF/HF, we found that the inverted-U curve remained significant with the interpolated maximum flow occuring when LF/HF balance was 6.822. Stated another way, the optimal flow experience occurred when the autonomic state comprised of 87% sympathetic and 13% parasympathetic response.

Our results also support the potential existence of an optimal autonomic state for high cognitive performance. Reaction time during the Stroop test is often referenced as a marker of executive function (Egner and Hirsch, 2005). When interpreting the effect of HRV on reaction times, the overall sympathovagal balance suggested the minimum reaction time at a sympathetically dominated balance. With the estimated minimum reaction time at LF/HF 11.61, this could be interpreted as the fastest reaction times occurring when the autonomic state is at predominantly (92%) sympathetic response. It should also be noted that while our results were generally statistically significant, autonomic state only explained approximately 10–20% of the overall variability observed in flow scores. While not able to fully account for changes in flow, our results suggest that autonomic state at least partially influences the flow experience. Other unaccounted factors, such as mindset or task familiarity, may play important roles in further determining the flow relationship.

The finding of increased arousal in autonomic states correlating with improved task performance is supported by several studies. In a study on executive function using a similar Stroop color test, increased sympathetic tone was associated with faster response times in color naming (Mathewson et al., 2010). Other studies have demonstrated that invoking a higher stress response can also increase cognitive performance. In a study on perceived threat of electric shock, subjects with increased anxiety demonstrated faster reaction time in executive tasks under the threat of unpredictable shocks to the hand (Cantelon et al., 2018). In studies on physical exertion in athletes, higher stress responses from exertion were associated with faster reaction times (Luft et al., 2009; Murray and Russoniello, 2012).

### Limitations

There are two main limitations to the external validity of our pilot study. Since estrogen levels can affect HRV during the stress response, we chose to limit our participant enrollment to male participants in order to minimize hormonal variability. Therefore, we may have limited generalizability to females. Future studies should validate our findings in a female population. Secondly, our findings are limited based on a relatively small pilot study. Replication of these findings should be attempted in a future on a larger sample size.

Use of LF/HF as a measure of sympathovagal balance has been highly debated (Eckberg, 1997; Billman, 2013), but despite this challenge, recent studies on stress response still use LF/HF as a marker for sympathovagal balance (Lennartsson et al., 2016; Cao et al., 2019). von Rosenberg et al. (2017) has proposed a new two-dimensional method of LF/HF analysis to categorize mental and physical stresses. This new methodology might be promising for a future analysis in a larger confirmatory study.

## Conclusion

When reviewing both executive task function and flow experience, both indices appeared to be maximized at approximately 90% sympathetic state. To our knowledge, this is the first study to suggest U-shaped relationships existing simultaneously for both flow experience and executive task function, suggesting that optimal performance may be associated with predominant, but not total, sympathetic response. Our findings are based on a small pilot so the results should be approached with caution. However, they do provide a preliminary foundation to understand the practical applications of autonomic modulation to potentially enhance performance during high-stress situations.

## Data Availability

The datasets generated for this study are available on request to the corresponding author.

## Ethics Statement

This study was carried out in accordance with the recommendations of IRB of the Harvard T.H. Chan School of Public Health with written informed consent from all subjects. All subjects gave written informed consent in accordance with the Declaration of Helsinki. The protocol was approved by the IRB of the Harvard T.H. Chan School of Public Health.

## Author Contributions

MC originated the study design, conducted the experiments, and performed all study analyses. SK contributed to the study design. Both authors drafted, revised, and approved the manuscript.

## Conflict of Interest Statement

The authors declare that the research was conducted in the absence of any commercial or financial relationships that could be construed as a potential conflict of interest.

## Footnotes

1 http://cognitivefun.net

## References

[B1] Barbosa M. P. da Silva N. T. de Azevedo F. M. Pastre C. M. Vanderlei L. C. (2016). Comparison of polar(R) RS800G3 heart rate monitor with Polar(R) S810i and electrocardiogram to obtain the series of RR intervals and analysis of heart rate variability at rest. *Clin. Physiol. Funct. Imaging* 36 112–117. 10.1111/cpf.12203 25348547

[B2] Bian Y. Yang C. Gao F. Li H. Zhou S. Li H. (2016). A framework for physiological indicators of flow in VR games: construction and preliminary evaluation. *Pers. Ubiquitous Comput.* 20 821–832. 10.1007/s00779-016-0953-5

[B3] Billman G. E. (2013). The LF/HF ratio does not accurately measure cardiac sympatho-vagal balance. *Front. Physiol.* 4:26 10.3389/fphys.2013.00026 PMC3576706 23431279

[B4] Cantelon J. A. Giles G. E. Eddy M. D. Haga Z. Mahoney C. R. Taylor H. A. (2018). Exerting cognitive control under threat: interactive effects of physical and emotional stress. *Emotion* 15. 10.1037/emo0000509 [Epub ahead of print]. 30321039

[B5] Cao X. MacNaughton P. Cadet L. R. Cedeno-Laurent J. G. Flanigan S. Vallarino J. (2019). Heart rate variability and performance of commercial airline pilots during flight simulations. *Int. J. Environ. Res. Public Health* 16:237. 10.3390/ijerph16020237 30654438 PMC6352143

[B6] Chin M. S. Kales S. N. (2019). Understanding mind–body disciplines: a pilot study of breathing and dynamic muscle contraction on autonomic nervous system reactivity. *Stress and Health* 1–7. 10.1002/smi.2887 31347763 PMC8758201

[B7] Csikszentmihalyi M. (1975). *Beyond Boredom and Anxiety*, 1st Edn San Francisco, CA: Jossey-Bass Publishers, 231.

[B8] de Looff P. C. Cornet L. J. M. Embregts P. Nijman H. L. I. Didden H. C. M. (2018). Associations of sympathetic and parasympathetic activity in job stress and burnout: a systematic review. *PLoS One* 13:e0205741. 10.1371/journal.pone.0205741 30335812 PMC6193670

[B9] de Manzano O. Theorell T. Harmat L. Ullen F. (2010). The psychophysiology of flow during piano playing. *Emotion* 10 301–311. 10.1037/a0018432 20515220

[B10] Dyer F. N. (1973). The stroop phenomenon and its use in the stlldy of perceptual, cognitive, and response processes. *Mem. Cogn.* 1 106–120. 10.3758/bf03198078 24214501

[B11] Eckberg D. L. (1997). Sympathovagal balance: a critical appraisal. *Circulation* 96 3224–3232. 10.1161/01.cir.96.9.3224 9386196

[B12] Egner T. Hirsch J. (2005). The neural correlates and functional integration of cognitive control in a Stroop task. *Neuroimage* 24 539–547. 10.1016/j.neuroimage.2004.09.007 15627596

[B13] Gaggioli A. Cipresso P. Serino S. Riva G. (2013). Psychophysiological correlates of flow during daily activities. *Stud. Health Technol. Inform.* 191 65–69. 23792845

[B14] Giles D. Draper N. Neil W. (2016). Validity of the polar V800 heart rate monitor to measure RR intervals at rest. *Eur. J. Appl. Physiol.* 116 563–571. 10.1007/s00421-015-3303-9 26708360 PMC4751190

[B15] Goyal M. Singh S. Sibinga E. M. Gould N. F. Rowland-Seymour A. Sharma R. (2014). Meditation programs for psychological stress and well-being: a systematic review and meta-analysis. *JAMA Intern. Med.* 174 357–368. 10.1001/jamainternmed.2013.13018 24395196 PMC4142584

[B16] Jackson S. A. Martin A. J. Eklund R. C. (2008). Long and short measures of flow: the construct validity of the FSS-2, DFS-2, and new brief counterparts. *J. Sport Exerc. Psychol.* 30 561–587. 10.1123/jsep.30.5.561 18971512

[B17] Jerath R. Edry J. W. Barnes V. A. Jerath V. (2006). Physiology of long pranayamic breathing: neural respiratory elements may provide a mechanism that explains how slow deep breathing shifts the autonomic nervous system. *Med. Hypotheses* 67 566–571. 10.1016/j.mehy.2006.02.042 16624497

[B18] Kanthak M. K. Stalder T. Hill L. K. Thayer J. F. Penz M. Kirschbaum C. (2017). Autonomic dysregulation in burnout and depression: evidence for the central role of exhaustion. *Scand. J. Work Environ. Health* 43 475–484. 10.5271/sjweh.3647 28514792 PMC5788013

[B19] Keller J. Bless H. Blomann F. Kleinböhl D. (2011). Physiological aspects of flow experiences: skills-demand-compatibility effects on heart rate variability and salivary cortisol. *J. Exp. Soc. Psychol.* 47 849–852. 10.1016/j.jesp.2011.02.004

[B20] Kim H. G. Cheon E. J. Bai D. S. Lee Y. H. Koo B. H. (2018). Stress and heart rate variability: a meta-analysis and review of the literature. *Psychiatry Investig.* 15 235–245. 10.30773/pi.2017.08.17 29486547 PMC5900369

[B21] Koenig J. Thayer J. F. (2016). Sex differences in healthy human heart rate variability: a meta-analysis. *Neurosci. Biobehav. Rev.* 64 288–310. 10.1016/j.neubiorev.2016.03.007 26964804

[B22] Kozhevnikov M. Li Y. Wong S. Obana T. Amihai I. (2018). Do enhanced states exist? Boosting cognitive capacities through an action video-game. *Cognition* 173 93–105. 10.1016/j.cognition.2018.01.006 29367017

[B23] Laborde S. Mosley E. Thayer J. F. (2017). Heart rate variability and cardiac vagal tone in psychophysiological research - recommendations for experiment planning, data analysis, and data reporting. *Front. Psychol.* 8:213. 10.3389/fpsyg.2017.00213 28265249 PMC5316555

[B24] Lehrer P. Vaschillo E. Trost Z. France C. R. (2009). Effects of rhythmical muscle tension at 0.1Hz on cardiovascular resonance and the baroreflex. *Biol. Psychol.* 81 24–30. 10.1016/j.biopsycho.2009.01.003 19428965

[B25] Lehrer P. M. Vaschillo E. Vaschillo B. (2000). Resonant frequency biofeedback training to increase cardiac variability: rationale and manual for training. *Appl. Psychophysiol. Biofeedback* 25 177–191. 10999236 10.1023/a:1009554825745

[B26] Lennartsson A. K. Jonsdottir I. Sjors A. (2016). Low heart rate variability in patients with clinical burnout. *Int. J. Psychophysiol.* 110 171–178. 10.1016/j.ijpsycho.2016.08.005 27535344

[B27] Luft C. D. Takase E. Darby D. (2009). Heart rate variability and cognitive function: effects of physical effort. *Biol. Psychol.* 82 164–168. 10.1016/j.biopsycho.2009.07.007 19632295

[B28] Malliani A. Lombardi F. Pagani M. (1994). Power spectrum analysis of heart rate variability: a tool to explore neural regulatory mechanisms. *Br. Heart J.* 71 1–2. 10.1136/hrt.71.1.1 8297682 PMC483598

[B29] Mathewson K. J. Jetha M. K. Drmic I. E. Bryson S. E. Goldberg J. O. Hall G. B. (2010). Autonomic predictors of Stroop performance in young and middle-aged adults. *Int. J. Psychophysiol.* 76 123–129. 10.1016/j.ijpsycho.2010.02.007 20193717

[B30] May R. W. Seibert G. S. Sanchez-Gonzalez M. A. Fincham F. D. (2018). School burnout and heart rate variability: risk of cardiovascular disease and hypertension in young adult females. *Stress* 21 211–216. 10.1080/10253890.2018.1433161 29382258

[B31] McCraty R. Shaffer F. (2015). Heart rate variability: new perspectives on physiological mechanisms, assessment of self-regulatory capacity, and health risk. *Glob. Adv. Health Med.* 4 46–61. 10.7453/gahmj.2014.073 25694852 PMC4311559

[B32] Motulsky H. J. Brown R. E. (2006). Detecting outliers when fitting data with nonlinear regression - a new method based on robust nonlinear regression and the false discovery rate. *BMC Bioinform.* 7:123. 10.1186/1471-2105-7-123 16526949 PMC1472692

[B33] Murray N. P. Russoniello C. (2012). Acute physical activity on cognitive function: a heart rate variability examination. *Appl. Psychophysiol. Biofeedback* 37 219–227. 10.1007/s10484-012-9196-z 22543813

[B34] Nijjar P. S. Puppala V. K. Dickinson O. Duval S. Duprez D. Kreitzer M. J. (2014). Modulation of the autonomic nervous system assessed through heart rate variability by a mindfulness based stress reduction program. *Int. J. Cardiol.* 177 557–559. 10.1016/j.ijcard.2014.08.116 25179555

[B35] Task Force of the European Society of Cardiology and the North American Society of Pacing and Electrophysiology (1996). Heart rate variability. Standards of measurement physiological. interpretation, and clinical use. *Eur. Heart J.* 17 354–381. 10.1093/oxfordjournals.eurheartj.a014868 8737210

[B36] Pagani M. Lombardi F. Guzzetti S. Rimoldi O. Furlan R. Pizzinelli P. (1986). Power spectral analysis of heart rate and arterial pressure variabilities as a marker of sympatho-vagal interaction in man and conscious dog. *Circ. Res.* 59 178–193. 10.1161/01.res.59.2.178 2874900

[B37] Peifer C. Schulz A. Schächinger H. Baumann N. Antoni C. H. (2014). The relation of flow-experience and physiological arousal under stress — Can u shape it? *J. Exp. Soc. Psychol.* 53 62–69. 10.1016/j.jesp.2014.01.009

[B38] Salahuddin L. Cho J. Jeong M. G. Kim D. (2007). Ultra short term analysis of heart rate variability for monitoring mental stress in mobile settings. *Conf. Proc. IEEE Eng. Med. Biol. Soc.* 2007 4656–4659. 10.1109/IEMBS.2007.4353378 18003044

[B39] Sato N. Miyake S. (2004). Cardiovascular reactivity to mental stress: relationship with menstrual cycle and gender. *J. Physiol. Anthropol. Appl. Hum. Sci.* 23 215–223. 10.2114/jpa.23.215 15599065

[B40] Shaffer F. Ginsberg J. P. (2017). An overview of heart rate variability metrics and norms. *Front. Public Health* 5:258. 10.3389/fpubh.2017.00258 29034226 PMC5624990

[B41] Sullivan M. B. Erb M. Schmalzl L. Moonaz S. Noggle Taylor J. Porges S. W. (2018). Yoga therapy and polyvagal theory: the convergence of traditional wisdom and contemporary neuroscience for self-regulation and resilience. *Front. Hum. Neurosci.* 12:67. 10.3389/fnhum.2018.00067 29535617 PMC5835127

[B42] Thayer J. F. Ahs F. Fredrikson M. Sollers J. J. III Wager T. D. (2012). A meta-analysis of heart rate variability and neuroimaging studies: implications for heart rate variability as a marker of stress and health. *Neurosci. Biobehav. Rev.* 36 747–756. 10.1016/j.neubiorev.2011.11.009 22178086

[B43] Traunmuller C. Stefitz R. Gaisbachgrabner K. Hofmann P. Roessler A. Schwerdtfeger A. R. (2019). Psychophysiological concomitants of burnout: evidence for different subtypes. *J. Psychosom. Res.* 118 41–48. 10.1016/j.jpsychores.2019.01.009 30782353

[B44] van den Broek P. Lorch R. F. Jr. Linderholm T. Gustafson M. (2001). The effects of readers’ goals on inference generation and memory for texts. *Mem. Cogn.* 29 1081–1087. 10.3758/bf03206376 11913743

[B45] Vaschillo E. G. Vaschillo B. Pandina R. J. Bates M. E. (2011). Resonances in the cardiovascular system caused by rhythmical muscle tension. *Psychophysiology* 48 927–936. 10.1111/j.1469-8986.2010.01156.x 21143610 PMC3094735

[B46] Visnovcova Z. Mestanik M. Javorka M. Mokra D. Gala M. Jurko A. (2014). Complexity and time asymmetry of heart rate variability are altered in acute mental stress. *Physiol. Meas.* 35 1319–1334. 10.1088/0967-3334/35/7/1319 24854052

[B47] von Rosenberg W. Chanwimalueang T. Adjei T. Jaffer U. Goverdovsky V. Mandic D. P. (2017). Resolving ambiguities in the LF/HF ratio: LF-HF scatter plots for the categorization of mental and physical stress from HRV. *Front. Physiol.* 8:360 10.3389/fphys.2017.00360 PMC5469891 28659811

[B48] Walther A. Lacker T. J. Ehlert U. (2018). Everybody was Kung-Fu fighting-The beneficial effects of Tai Chi Qigong and self-defense Kung-Fu training on psychological and endocrine health in middle aged and older men. *Complement Ther. Med.* 36 68–72. 10.1016/j.ctim.2017.11.021 29458935

[B49] Zhang M. Liu L. Shi Y. Yang Y. Yu X. Angerer P. (2018). Longitudinal associations of burnout with heart rate variability in patients following acute coronary syndrome: a one-year follow-up study. *Gen. Hosp. Psychiatry* 53 59–64. 10.1016/j.genhosppsych.2018.05.008 29859340

[B50] Zimmermann-Viehoff F. Thayer J. Koenig J. Herrmann C. Weber C. S. Deter H. C. (2016). Short-term effects of espresso coffee on heart rate variability and blood pressure in habitual and non-habitual coffee consumers–a randomized crossover study. *Nutr. Neurosci.* 19 169–175. 10.1179/1476830515Y.0000000018 25850440

